# Supplementary material for: A dual role for PGLYRP1 in host defense and immune regulation during B. pertussis infection
Source: eLife. 2026 Aug 11;15:RP108947. doi: 10.7554/eLife.108947 (PMC13461146; doi:10.7554/eLife.108947)
Supplement: Peer review history

# Editor report: eLife Assessment

**DOI:** 10.7554/eLife.108947.3.sa0

**Authors:** Joseph P Dillard

**Affiliations:** University of Wisconsin-Madison United States

**Keywords:** Solid, Useful

Rickert and colleagues demonstrate that the host peptidoglycan-binding protein PGLYRP1 has both beneficial and detrimental effects on Bordetella pertussis infection in mice. Using a **solid** array of techniques, the study provides **useful** insights into how the peptidoglycan fragment tracheal cytotoxin alters host immune responses, dampening inflammatory responses later in B. pertussis infection. These studies indicate that release of peptidoglycan fragments with particular structures can be used by bacteria to modulate NOD1 versus NOD2 responses to their advantage.

# Referee report: Reviewer #1 (Public review)

**DOI:** 10.7554/eLife.108947.3.sa1

Summary:

The authors aim to demonstrate that PGLYRP1 plays a dual role in host responses to B. pertussis infection. PGLYRP1 signaling is known to activate bactericidal responses due to recognition of peptidoglycan. Through NOD1 activation and TREM-1 engagement, it appears PGLYRP1 also has immunomodulator activities. The authors present mouse knockout studies and gene expression data to illustrate the role of PGGLYRL1 in relation to B. pertussis peptidogylcan. Mice lacking PGLYRP1 had slightly lower pathology scores. When TCT peptidoglycan was removed from the bacteria, surprisingly IL23A, IL6, IL1B and other pro-inflammatory genes encoding cytokines increased. The relationship to TCT and PGLYRP1 suggest the pathogen uses this strategy to decrease immune activation. The authors when on to show the relationship between PGLRP1 and TREM-1 as mediated with PGN using various versions of peptidoglycan. The study presents multiple angles of data to back up its findings and demonstrates an interesting strategy used by B. pertussis to down-regulate innate responses to its presence during infection.

Strengths:

Use of knockout mice of the key factor being considered paired with isogenic B. pertussis strains to reveal the mechanism of immune modulation to benefit the bacteria. The authors used in vivo gene expression paired with in vivo assays to establish each aspect of the mechanism.

Weaknesses:

The main focus was on innate responses, but some analysis of antigen specific antibody responses could improve the impact of the findings.

Comments on revised version.

I have no further input to add.

# Referee report: Reviewer #2 (Public review)

**DOI:** 10.7554/eLife.108947.3.sa2

Since its original discovery, the mechanistic basis for TCT-mediated pathogenesis of Bordetella pertussis has been a moving target and difficult to uncouple from confounding variables. The current study provides some exciting data that suggest PGLYRP-1 modulates host responses upon 'activation' by TCT. While there are some strengths associated with the unbiased approaches and collective data to support the claims associated with TCT and PGLYRP-1's function in this system, caution should be used when interpreting and extrapolating some the information provided. While many of the initial concerns were addressed, one concern remains: using whole, intact PG sacculi from other species for comparative studies with a fragment of released PG (i.e., TCT).

Comments on revised version.

I have no further comments.

# Referee report: Reviewer #3 (Public review)

**DOI:** 10.7554/eLife.108947.3.sa3

Summary:

This study evaluates the contributions of the mammalian PG-binding protein PGLYRP1 to Bordetella infection. The authors find potential roles for PGLYRP1 in both bacterial killing (canonical) and regulation of inflammation (non-canonical). While these are interesting findings and the idea that PG fragment release has differential impacts on infection depending on fragment structure, the study is ultimately limited by the lack of connection between the in vivo and in vitro experiments and determining the precise mechanism of how PGLYRP1 regulates host responses and bacterial fitness during infection requires further study.

Strengths:

(1) The combination of scRNAseq with in vitro and in vivo assays provides complementary views of PGLYRP1 function during infection.

(2) The use of TCT-deficient B. pertussis provides a useful control and perturbation in the in vitro assays.

Weaknesses/Areas for future study:

(1) The study does not ultimately resolve the initial early versus late phenotype divergence. While the in vitro assays suggest explanations for their in vivo observations, further mechanistic links are lacking and necessary for the author's conclusions throughout. To state one example, what is the early and late infection phenotype of TCT- Bp in mice lacking PGLYRP1? RNAseq data is reported from these mice but there are no burden or pathology studies. Furthermore, what are the neutrophil phenotypes (NOD-1/TREM-1 activation) in vivo? And are they dependent on PGLYRP1 and/or TCT? This will be an important topic of future study, as noted by the authors in their response.

(2) It is unclear whether or how the NOD1 and TREM-1 pathways interact.

(3) Many of the study's conclusions rely on the use of HEK293 reporter lines in the absence of bacterial infection, which may not be physiologically representative.

Comments on revised version.

The authors have responded adequately to my comments.

# Author comment: Author response

**DOI:** 10.7554/eLife.108947.3.sa4

**Authors:** David M Rickert, Sasha Cardozo, Nicholas Carbonetti, William E Goldman, Karen M Scanlon, Ciaran Skerry

**Affiliations:** University of Maryland, Baltimore Baltimore United States; University of Maryland, Baltimore Baltimore United States; University of Maryland, Baltimore Baltimore United States; University of North Carolina at Chapel Hill Chapel Hill United States; University of Maryland, Baltimore Baltimore United States; University of Maryland, Baltimore Baltimore United States

The following is the authors’ response to the original reviews.

**Reviewer #1 (Public review):**

Summary:

The authors aim to demonstrate that PGLYRP1 plays a dual role in host responses to B. pertussis infection. PGLYRP1 signaling is known to activate bactericidal responses due to recognition of peptidoglycan. Through NOD1 activation and TREM-1 engagement, it appears PGLYRP1 also has immunomodulator activities. The authors present mouse knockout studies and gene expression data to illustrate the role of PGLYRP1 in relation to B. pertussis peptidoglycan. Mice lacking PGLYRP1 had slightly lower pathology scores. When TCT peptidoglycan was removed from the bacteria, surprisingly IL23A, IL6, IL1B, and other pro-inflammatory genes encoding cytokines increased. The relationship to TCT and PGLYRP1 suggests the pathogen uses this strategy to decrease immune activation. The authors went on to show the relationship between PGLRP1 and TREM-1 as mediated by PGN using various versions of peptidoglycan. The study presents multiple angles of data to back up its findings and demonstrates an interesting strategy used by B. pertussis to downregulate innate responses to its presence during infection.

Strengths:

Use of knockout mice of the key factor being considered, paired with isogenic B.

pertussis strains, to reveal the mechanism of immune modulation to benefit the bacteria. The authors used in vivo gene expression paired with in vivo assays to establish each aspect of the mechanism.

Weaknesses:

The main focus was on innate responses, and some analysis of antigen-specific antibody responses could improve the impact of the findings.

The authors thank the reviewer for their careful reading of the manuscript. We agree that understanding the impact of peptidoglycan recognition in adaptive immunity, including antibody responses, would be beneficial. This is particularly apparent due to the pressing need for novel vaccination strategies for pertussis. To this end, we have modified the discussion section to highlight this and are embarking on detailed studies of the adaptive response generated with B. pertussis strains releasing alternative peptidoglycan structures.

**Reviewer #1 (Recommendations for the authors):**

(1) This reviewer is of the opinion that describing the PGLYRP1 as a "bactericidal protein" seems misleading. "To determine whether PGLYRP1 has bactericidal activity against B. pertussis, we performed in vitro and ex vivo killing assays." Bactericidal activity was measured in normal or knockout neutrophils, but this seems to say the PGLYRP1 itself is an antimicrobial peptide. It clearly plays a role in the response,e but it is a regulator and not a killing agent.

We agree that ‘bactericidal’ is not the most accurate description and have revised the manuscript accordingly to be more accurate throughout results section 1.

(2) PGN can induce IgM production. Antibody production of any type was absent from this study. Would IgA/IgM/IgG levels to B. pertussis or its TCT change due to PGLYRP1? To this reviewer, it would be good to use the serum and perform some ELISA analysis. It is also likely that T cell responses could be impaired, but that may be out of the scope of this manuscript, but could be acceptable to consider for future studies.

The authors thank the reviewers for this suggestion. We have added text to the discussion section to highlight the importance and potential of this suggestion.

(3) Please include sources of mice (vendors) and strain numbers for transparency.

The authors have added the relevant detail to the methods section to address this valid concern.

(4) Were female or male mice or both used?

For PGLYRP1 vs BALB/c comparisons both male and female mice were used. These are presented as combined data. No discernible differences were noted between male and female mice following infection. For single cell RNA sequencing studies only female mice were used, to be consistent with the published pertussis mouse model and avoid sex-based complications in analysis. We have clarified these details in the text.

(5) It appears B. pertussis was cultured in SSM or BG. What condition was used for the bacteria used for the mouse challenge? SSM or BG?

For mouse studies, bacteria were grown on BG agar supplemented with 10% defibrinated sheep blood for 48 hours and inoculum prepared by suspending in PBS in accordance with our established protocols. For in vitro studies liquid cultures were grown to mid-log in SSM. This has now been clarified in the methods section

(6) Are the raw RNAseq and scRNAseq reads deposited in SRA?

Raw data has now been deposited in the Gene Expression Omnibus (GEO) under number GSE324217

(7) Is the scRNAseq data from one mouse or a pooled set of mice? If pooled, were the individual mice barcoded?

scRNAseq data was obtained from barcoded individual samples and replicates were pooled and integrated during analysis, but the individual mouse each cell came from is still noted in the downstream analysis. This is now clarified in methods.

(8) Why were some studies done by aerosol and others were done by intranasal delivery?

The authors thank the reviewer for careful reading of the manuscript which erroneously listed aerosol infections. All infections in these studies were intranasal. This has now been rectified in the text.

**Reviewer #2 (Public review):**

Since its original discovery, the mechanistic basis for TCT-mediated pathogenesis of Bordetella pertussis has been a moving target and difficult to uncouple from confounding variables. The current study provides some exciting data that suggest PGLYRP-1 modulates host responses upon 'activation' by TCT. While there are some strengths associated with the unbiased approaches and collective data to support the claims associated with TCT and PGLYRP-1's function in this system, caution should be used when interpreting and extrapolating some of the information provided. For instance, the amount and purity of TCT used in the studies are unclear, and the in vitro activity of PGLYRP1 on B. pertussis is questionable. Different mouse backgrounds are used for various assays throughout, and it is known that the PRRs vary in these systems, so the confounding variables are difficult to uncouple. Additional concerns include the types of statistical tests being performed to support some of the claims and the relevance of using whole, intact PG sacculi from other species for comparative studies with a fragment of released PG (i.e., TCT).

We thank the reviewer for their insightful suggestions to improve the standard of our manuscript and for highlighting several important considerations regarding our interpretation of TCT mediated host responses. We have addressed the points made in the revised manuscript. In particular, we have amended the Methods section to include a description of the purification and quantification of tracheal cytotoxin. These additions clarify the dosing of TCT used throughout the manuscript. We have revised the Results and Discussion sections to avoid overstating the bactericidal activity of PGLYRP1 against B. pertussis and to more carefully describe in vitro observations. Our revised interpretation emphasizes the role of PGLYRP1 in modulating host immune responses. Additionally, we have clarified experimental design and strain usage descriptions in the Methods section. The reviewer provided valuable and insightful comments on the solubility and structure of muropeptides studies. In response, we have revised the Results and Discussion sections to acknowledge these differences and the limitations they pose. Further, we have removed conclusions regarding the specific role of the 1,6anhydro bond. The statistical analyses have been reviewed and validated as well as clarified throughout the manuscript and Methods and figure legends updated.

We appreciate the reviewer’s comments and believe the revisions have improved the clarity and rigor of the manuscript while maintaining the central conclusions about how peptidoglycan recognition influences host inflammatory responses during B. pertussis infection.

**Reviewer #2 (Recommendations for the authors):**

Major Points:

(1) The concentration, purity, etc. of TCT seems like it is entirely unknown. Only a couple of experiments actually state the amount used, and it's unclear how the author determined the concentration because this is not trivial. Given the long-standing concerns with purity and co-purifying contaminants, this issue is paramount and needs to be properly addressed.

TCT was purified by HPLC in the Goldman lab (UNC). Concentration was determined by comparing the peak area of each preparation to a purified TCT standard quantified by amino acid analysis. We have added these details to the Methods and now report concentrations throughout the manuscript.

(2) Related to the effects of bacterial PG, studies performed are comparing TCT (a muropeptide) to commercially acquired, insoluble PG sacculi from *B. subtilis* and *S. aureus*. One cannot make these comparisons. There are flaws in terms of solubility (one goes into solution, the other does not), the amount used, the molar concentrations, etc. The authors also state that these are non-1,6 anhydro PG samples. That is not true. They contain plenty of 1,6 anhydroMurNAc, the moiety just exists in a different form. Finally, *B. subtilis* PG is not just mDAP, it's amidated, which is known to have effects on host response(s).

We thank the reviewer for this important critique. We agree that differences in solubility and structural composition between TCT and PG sacculi limit direct comparisons. We have revised the Results and Discussion to remove statements implying direct equivalence and instead frame these experiments as highlighting how structural and physical properties of PGN fragments influence PGLYRP1-mediated activation of TREM-1. We have also removed statements regarding the 1,6-anhydro bond which were not adequately supported.

(3) The claim that PGLYRP-1 is bactericidal in vitro is not supported by the data. Figure 1G shows that 24 hours after incubation, there is no difference. The comparison is being made to BSA, which is much higher (possibly because they're catabolizing it?) and thus entirely inappropriate. All other data in Figure 1 suggest no effect in vitro. In fact, it's this reviewer's position that none of the studies in Figures 1G, H, and I are convincing and should be entirely excluded.

The authors agree that language describing the bactericidal assays is not optimal and have made revisions. The text in this results section has been modified to more carefully describe bacterial killing assays and accurately describe the effects the data suggest, primarily removing claims of bactericidal effects. BSA was chosen as a control protein (concentration matched with PGLYRP1), based on published controls for PGLYRP bactericidal assays (Lu et al., 2006, JBC) similar results were obtained with PBS (volume matched with PGLYRP1). Descriptions of Fig1G,H,I have been updated. Data in 1H demonstrates that TCT release does not protect against effects of PGLYRP1, despite free PGN inhibiting PGLYRP1 bactericidal activity in published literature, while 1I suggests that extracellular polysaccharides contribute to protection against PGLYRP1 activity, preventing a more bactericidal phenotype which were not observed in the earlier assays when B. pertussis retained its capacity to produce bps polysaccharide.

(4) Histology studies are unclear, and the data presented do not support the claims. Not only are the methods and results text describing the analysis contradictory, but nowhere are the actual statistical tests supporting the claims that they are different provided. This might be an oversight, but based on the variation, I would be surprised if they were statistically significantly different if proper tests are being used.

Significance for pathology scores were initially determined using 2-way ANOVA as we had 4 groups (WT&KO at 4&7DPI) providing p-values of 0.01 for WT vs KO at 7DPI and 0.003 at 4DPI. Following reviewers’ suggestions, we have reanalyzed these data using a Mann Whitney U test, which is more appropriate for comparisons between two groups. This analysis yielded p-values of 0.013 (4DPI) and 0.00316 (7DPI) respectively confirming that the observed differences remain statistically significant. Statistical methods are now described in the methods and figure legends.

(5) The NOD reporter studies are not well controlled and should include (a) mouse vs human for both NOD1 and NOD2; (b) defined details in terms of how spent culture media was treated, amount of material normalized, etc., (c) concentrations of all materials used.

We appreciate the reviewer’s comments regarding the NOD reporter assays. In response: (a) We have clarified and articulated the murine/human NOD reporter assays and included both human and mouse NOD1, along with controls. (b) We have supplemented descriptions of how conditioned (spent) culture media were collected, processed, and normalized in the ‘Bacterial strains and infections’ and ‘Reporter Cell Assays’ methods sections; (c) and the final concentrations of all agonists and test materials used in the reporter assays are now specified in the Methods and corresponding figure legends. Together, these additions address the requested controls and clarify the experimental conditions

(6) The scRNA-seq studies are provocative and informative, but the data shown are selectively included for the purposes of the paper. This is justified in terms of 'telling a story', but it's a disservice to the community not to include all the raw data attained. These should be deposited in an open-source system.

The complete dataset has now been deposited in GEO (GSE324217) enabling full access for the community. The analyses presented in the manuscript focus on the datasets most relevant to the central conclusions.

Minor points:

(1) The authors refer to arthropod PGRPs but call them PGLYRPs. It is best to stick with the established nomenclature and use the proper names to distinguish each. There are a few sentences in the abstract that don't make sense as they're written.

The authors thank the reviewer for their careful reading of the manuscript and have altered the manuscript to use PGRP for arthropod peptidoglycan recognition proteins.

(2) The reciprocal result of bacterial burden at different time points in the context of PGLYRP-1 production in mice could be simply explained - it is bactericidal early, and the accumulation of dead/dying bacteria releases large pieces of PG that are not released during growth (anhydro) but rather lysis. It is the latter that causes the inverse relationship later.

The authors believe this is an interesting and plausible explanation for differences in responses at different stages of disease. Further, we believe that elucidating the mechanism by which ‘large pieces of PG not released during growth” are recognized differently than PG from lysed bacteria is worthwhile. We speculate that the release of TCT could be a mechanism by which B. pertussis takes advantage of host differences in PG recognition. We thank the reviewers for this thought and have included this possible interpretation in the text.

(3) The results section references Figure 1G while discussing results presented in Figure 1H.

This has now been corrected.

**Reviewer #3 (Public review):**

Summary:

This study evaluates the contributions of the mammalian PG-binding protein PGLYRP1 to Bordetella infection. The authors find potential roles for PGLYRP1 in both bacterial killing (canonical) and regulation of inflammation (non-canonical). While these are interesting findings and the idea that PG fragment release has differential impacts on infection depending on fragment structure, the study is limited by the lack of connection between the in vivo and in vitro experiments, and determining the precise mechanism of how PGLYRP1 regulates host responses and bacterial fitness during infection requires further study.

Strengths:

(1) The combination of scRNAseq with in vitro and in vivo assays provides complementary views of PGLYRP1 function during infection.

(2) The use of TCT-deficient B. pertussis provides a useful control and perturbation in the in vitro assays.

Weaknesses:

(1) The study does not ultimately resolve the initial early versus late phenotype divergence. While the in vitro assays suggest explanations for their in vivo observations, further mechanistic links are lacking and necessary for the author's conclusions throughout. To state one example, what is the early and late infection phenotype of TCT- Bp in mice lacking PGLYRP1? RNAseq data are reported from these mice, but there are no burden or pathology studies. Furthermore, what are the neutrophil phenotypes (NOD-1/TREM-1 activation) in vivo? And are they dependent on PGLYRP1 and/or TCT?

(2) It is unclear whether or how the NOD1 and TREM-1 pathways interact.

(3) Many of the study's conclusions rely on the use of HEK293 reporter lines in the absence of bacterial infection, which may not be physiologically representative.

(4) The methods lack detail overall, and the experimental procedures should be described more concretely, especially for the scRNAseq datasets.

We thank the reviewer for their comprehensive and fair assessment of our study and for highlighting both its strengths and areas where clarification could improve the manuscript. As noted in the review the possibility that peptidoglycan fragment structure impacts disease pathogenesis is interesting and the role of PGLYRP1 in regulating host and bacterial fitness during infection requires further study.

We have addressed the points made by the reviewer in the revised manuscript. We edited the Methods section to provide additional experimental detail, particularly for the scRNA-seq analyses and reporter assays. We also clarified the experimental design and interpretation of the in vitro studies to avoid overstating mechanistic conclusions.

Studies with TREM-1 and NOD are attempting to assess multiple aspects of PGN/PGLYRP mediated enhancement of inflammatory responses via NFkB/MAPKs. No attempts have been made to assess synergistic, overlapping or compensatory effects between these systems. Other work from our group highlights the role of peptidoglycan in driving inflammatory responses via NOD receptors (doi: https://doi.org/10.1101/2025.08.08.669383) and TREM-1 (doi: 10.1128/IAI.00126-21). Work in this paper assesses the contribution of these pathways to the observed immune modulation noted by PGLYRP1.

We have clarified figure legends and analyses, including interpretation of neutrophil transcriptional programs identified in scRNAseq datasets and comparisons to known neutrophil phenotypes.

We appreciate the reviewers feedback and the opportunity to improve the clarity of our manuscript and optimize the conclusions and central findings.

**Reviewer #3 (Recommendations for the authors):**

(1) Please clarify in Figure 1C what the axis means, since the text refers to both uninfected and infected cells. What data allow the conclusion that PGLYRP1 expression "expanded" to other cell subsets?

We thank the reviewers for catching this oversight. We were relying on data which we had not best represented in Figure 1C, so we updated this figure and corresponding text so that this violin plot demonstrates increased PGLYRP1 expression levels and an increasing or expanding number of cell types following infection. This is now also reflected in the text. Expression of PGLYRP1 is apparent in more cell types and to a greater extent following infection (red) with B. pertussis compared to PBS challenge (black). Expression represents normalized and transformed unique molecular identifier counts per gene per cell.

(2) Please revise the Figure 1 legend to match the Figure panels, and mention the time point of the mPGLYRP1 killing assay in 1H/I. Were these assays performed at 6 or 24 hours? This could affect the interpretation of the data.

This has been revised to reflect timing of data.

(3) The text at the end of the first Results section is overstated, as the data in Figure 1 do not relate to immune-mediated clearance apart from expression levels.

This text has been revised and reference to immune mediated clearance removed

(4) More detail is needed in the explanation of Figures 3E-G. Do the neutrophil subsets correspond to known subsets from the literature?

When we overlaid established neutrophil signatures from the literature onto our dataset the NOD2+ neutrophils most closely resembled inflammatory or activated neutrophil programs described previously (Xie et al. 2020 Nat. Immuno., Veglia et al. 2021 J. Exp. Med)- specifically, high il1a, Ccl3 and Ptgs2 expression. In contrast, NOD1+ neutrophils showed greater overlap with resolving or regulatory neutrophil states- including genes associated with lipid mediator metabolism and NFkB dampening. Importantly, the clustering itself was not driven by NOD1 or NOD2 expression alone. NOD expression segregated within transcriptionally distinct neutrophil programs that are consistent with previously described inflammatory versus regulatory subsets. We included descriptions of these inflammatory neutrophils and related them to previously identified neutrophil populations, supporting our findings and improving the representation and articulation of the single cell neutrophil data analysis. We deeply thank the reviewers for their help in improving this section.

(5) The Methods section describes qPCR, but this is not presented in the Results.

This has now been removed. We thank the reviewer for their careful and complete review of the manuscript.
